# BCP-ALL blasts are not dependent on CD19 expression for leukaemic maintenance

**DOI:** 10.1038/leu.2016.64

**Published:** 2016-04-08

**Authors:** J Weiland, D Pal, M Case, J Irving, F Ponthan, S Koschmieder, O Heidenreich, A von Stackelberg, C Eckert, J Vormoor, A Elder

**Affiliations:** 1 Northern Institute for Cancer Research, Newcastle Cancer Centre, Newcastle University, Newcastle upon Tyne, UK; 2 Department of Hematology, Oncology, Hemostaseology, and Stem Cell Transplantation, Faculty of Medicine, RWTH Aachen University, Aachen, Germany; 3 Department of Paediatric Oncology/Haematology, Charité—Universitätsmedizin Berlin, Berlin, Germany; 4 Great North Children's Hospital, Newcastle upon Tyne Hospitals NHS Foundation Trust, Newcastle upon Tyne, UK

In recent years the anti-CD19/anti-CD3 bispecific antibody blinatumomab and chimeric antigen receptor (CAR) modified T cells targeting CD19 have shown early efficacy in clinical trials of paediatric and adult B-cell precursor acute lymphoblastic leukaemia (BCP-ALL).^1, 2, 3, 4, 5^ The rationale behind targeting CD19 in BCP-ALL is primarily its homogenous cell surface expression and B-lineage specificity.^5^ Thus, the entire malignant cell population should be targeted and eradicated by anti-CD19-directed immunotherapies. CD19 would be expected to have important functions in BCP-ALL survival, based on its roles in enhancing pre-B-cell receptor (pre-BCR) mediated phosphoinositide 3-kinase (PI3K) signalling and through pre-BCR-independent pathways such as MYC activation.^6, 7, 8, 9^ However, the effects of CD19 depletion on BCP-ALL cells have not been investigated. Hence, we examined the role of CD19 in leukaemic maintenance by silencing its expression in BCP-ALL cell lines and primograft samples using RNA interference.

First, we explored the effects of CD19 knockdown in CD19^+^ BCP-ALL cell lines reflecting three maturation stages of BCP-ALL: pro-B-ALL SEM (CD10^−^CD19^+^), common B-ALL REH (CD10^+^CD19^+^) and pre-B-ALL 697 (CD10^+^CD19^+^cyIgM^+^). BCP-ALL cell lines were transduced with two different lentiviral constructs targeting CD19. A short hairpin RNA (shRNA) that specifically targets the fusion gene *RUNX1/ETO* without affecting endogenous RUNX1 was used as a control,^10^ as this fusion is not found in any of the cell lines or primografts used (see Supplementary Methods for further details). We chose the pTRIPZ system, which allows doxycycline-mediated induction of shRNA) expression and puromycin selection of successfully transduced cells. Five days after induction, CD19 surface expression was reduced 15-fold in SEM cells, sixfold in REH cells and 14-fold in 697 cells by the best construct (shCD19II) compared to the geometric mean of expression in the control (Supplementary Figure 1). The CD19 depletion was maintained over several time points (Supplementary Figure 2). None of the cell lines showed any impairment in proliferation over 23–26 days (Figure 1a) or doubling times: SEM (control: 34 h vs shCD19: 33 h), REH (control: 38 h vs shCD19: 34 h) and 697 (control: 30 h vs shCD19: 29 h). This suggests that, at the level of knockdown achieved, CD19 is not essential for the proliferation of BCP-ALL cell lines in suspension culture.

We next considered that CD19 could be important for niche interactions, so we used a murine stromal cell feeder layer to mimic this. Cells were grown on M2-10B4 cells in medium containing 2% fetal bovine serum, which was the level at which the SEM and 697 cells developed dependence on the feeder layer for growth at low cell densities (10^4^ cells/ml) (Supplementary Figure 3). REH cells did not develop feeder dependence to the same extent as the other cell lines. Next, we performed a competitive assay under these conditions. We seeded equal mixtures of transduced and un-transduced cell populations to determine whether CD19-depleted cells were at a competitive disadvantage to wild-type cells. The ratio of RFP^+^ (shRNA-expressing) to RFP^−^ (wild-type) cells was used to assess changes in the proportions of each population. We did not observe any substantial differences in the ratio of CD19^−^ to wild-type cells for any of the three cell lines studied (Figure 1b), indicating that CD19 expression does not give cells a competitive growth advantage when adherent on stroma cells.

We also studied the effects of CD19 silencing in a high-risk pre-B-ALL t(17;19) primograft, L707, achieving a threefold reduction in CD19 expression using a constitutively active pGIPZ-shCD19 construct (Supplementary Figure 4). L707 cells were cultured on a human mesenchymal stem cell feeder layer and assessed for cell proliferation after puromycin selection. CD19-depleted L707 cells did not exhibit impaired growth compared to control cells (Figure 1c) and were not at a competitive disadvantage compared to control cells (Figure 1d). This suggests that CD19 is not required for leukaemic maintenance in primary blasts.

In support of our *in vitro* data, we obtained a patient sample taken at relapse (LK194), which presented as CD19^+^ BCP-ALL but relapsed as CD19^−^ BCP-ALL after treatment with blinatumomab (Figures 2a and b, Supplementary Figure 5). To investigate if these CD19^−^ cells could engraft and reconstitute leukaemia and if the CD19^−^ phenotype is stable *in vivo*, we injected 1 × 10^6^ CD19^−^ primary cells each into three NOD/LtSz-scid IL-2Rγ null (NSG) mice. All mice presented enlarged spleens (0.56–1.05 g, compared to <0.1 g for non-engrafted mice) and histological analysis showed leukaemic infiltration of the bone marrow (Supplementary Figure 6). Fluorescence-activated cell sorting analysis of harvested spleen samples showed that CD19^−^ blasts were able to engraft and reconstitute the leukaemia, and that the phenotype appeared stable (Figures 2c and e, Supplementary Figure 7). In one mouse (Figure 2d), a CD19^+^ population also emerged. These cells were also evident in CD19-stained histological sections (Supplementary Figures 6C and D). To investigate the relative repopulating ability of these populations, we re-transplanted the primograft into secondary recipients. In three secondary mice, the CD19^+^ peak appeared to increase in size relative to the CD19^−^ cells, suggesting the CD19^+^ blasts have a slight advantage in repopulating ability *in vivo* (Figure 2f, Supplementary Figure 8). However, a defined CD19^+^ population did not emerge in a secondary transplant of one of the CD19^−^ primografts (Figure 2g), demonstrating that the blasts are not dependent on CD19 for survival *in vivo*. It is unclear whether the CD19^+^ cells grew from a pre-existing minor CD19^+^ subclone in the relapsed sample, or arose from the CD19^−^ population through re-expression of CD19. A recent study has demonstrated exon 2 skipping as a mechanism of resistance to anti-CD19 therapeutics, whereby cells express a truncated CD19 protein to evade detection by anti-CD19 CAR T cells.^11^ Analysis of LK194 primograft using reverse transcriptase-PCR demonstrated expression of a transcript containing exons 1–3, with no evidence of exon 2 skipping (Figure 2h). Moreover, sequencing confirmed that the CD19 cDNA from LK194 was fully intact, compared with both a reference transcript and cDNA from the SEM cell line (Supplementary Figure 9). The presence of full-length CD19 transcript in these cells, but absence of protein, demonstrates evidence of an alternative mechanism of resistance to T-cell therapies to that previously described.^11^ Further investigation of the mechanisms by which this occurs will be important for understanding how to circumvent the development of resistance to anti-CD19 therapies in patients.

In summary, we have shown that BCP-ALL cell lines and primary blasts are not dependent on CD19 for survival and propagation in both *in vitro* and *in vivo* settings. This is contrary to the expectation given the functions of CD19 in pre-BCR and PI3K signalling and suggests that CD19-independent pathways play an important role in BCP-ALL development. Further investigation of the differences between CD19^+^ and CD19^−^ ALL blasts will be important to understand these pathways. Our data are consistent with other studies reporting CD19^−^ leukaemias^12, 13^ and demonstrating the emergence of CD19^−^ BCP-ALL clones following therapies targeting CD19.^3, 4^ Similarly, immature CD34^+^/CD19^−^ cells from primary high-risk paediatric ALL samples have previously been shown to engraft and maintain leukaemia in immunodeficient mice.^14^ It is therefore evident that leukaemic blasts that lose expression of the CD19 antigen will be able to survive and escape CD19-targeted therapies, increasing the risk of CD19^−^ relapse. It remains to be seen how big an impact this will have on the long-term efficacy of CD19 immunotherapies. Thus, in the future it will be vital to identify how best to integrate these immunotherapies into standard treatment protocols^15^ and develop combination therapies to eliminate emergent CD19^−^ blasts.

## Figures and Tables

**Figure 1:**
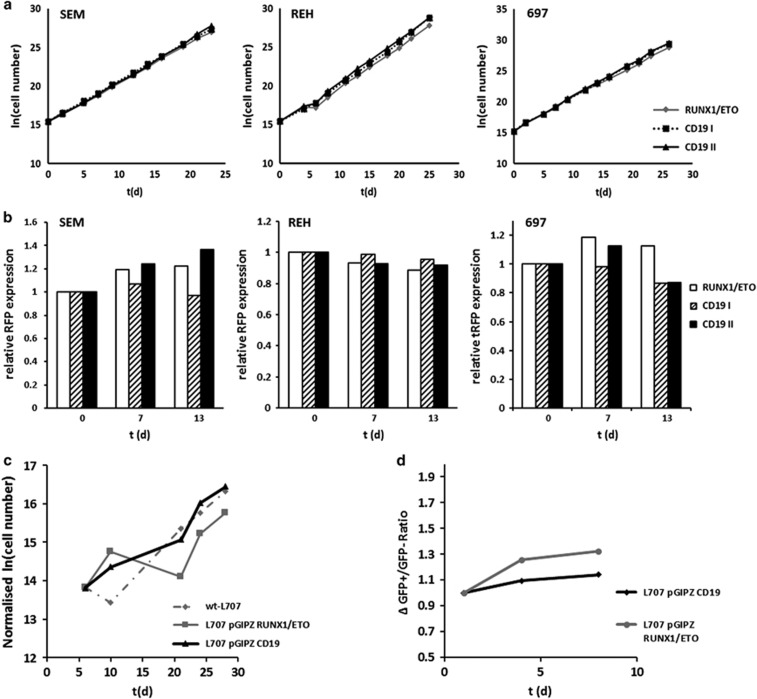
CD19 knockdown in BCP-ALL cell lines and high-risk BCP-ALL primograft does not affect cell growth. (**a**) Cell growth of BCP-ALL cell lines transduced with two different shRNA constructs targeting CD19 or RUNX1/ETO (control) in suspension culture. (**b**) CD19-depleted cells are not disadvantaged in a competitive setting. Populations of untransduced BCP-ALL cell lines and cells transduced with a pTRIPZ shRNA construct were mixed at low cellular density under serum-starved conditions on feeder cells. Relative tRFP expression represents the proportion of cells expressing the construct. The graph shows the mean of two independent experiments. (**c**) Cell growth of BCP-ALL primograft (L707) transduced with pGIPZ shRNA constructs against CD19 or RUNX1/ETO on human mesenchymal stem cell (hMSC) feeder cells in comparison with untransduced cells. Cell numbers were equalised at the first timepoint to allow comparison of growth rates. (**d**) Competitive assay of BCP-ALL primograft transduced with CD19 or RUNX1/ETO constructs mixed with untransduced BCP-ALL primograft on hMSC feeder cells. The graph shows the change in proportion of GFP-positive to -negative cells over the time course. The GFP-positive cells correspond to the fraction expressing the construct.

**Figure 2:**
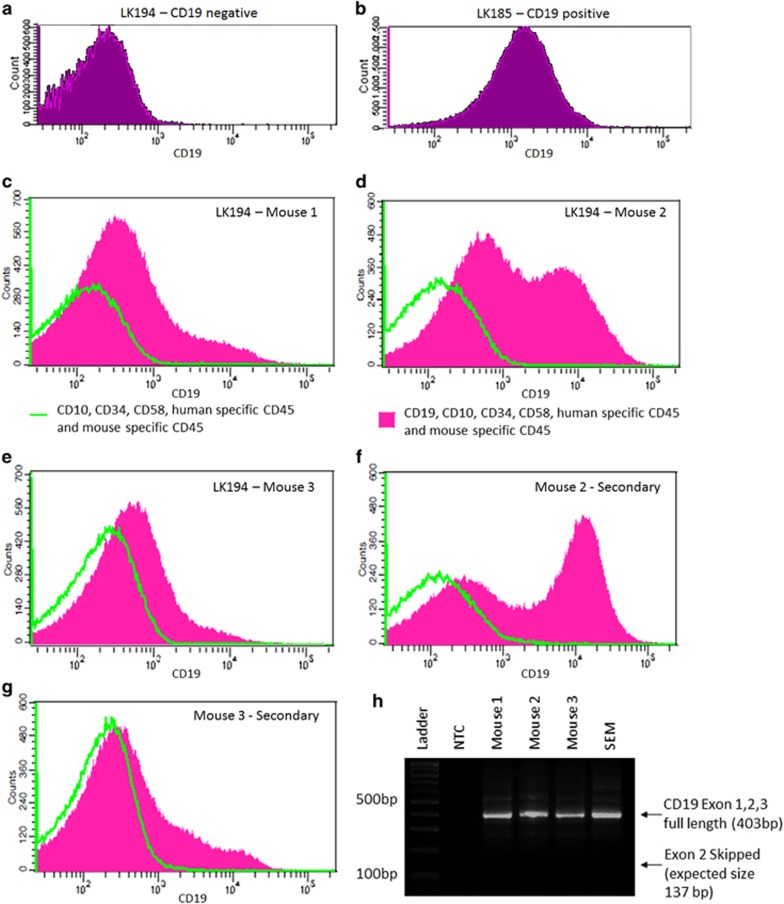
The CD19^−^ BCP-ALL sample is able to engraft NSG mice. Histogram showing CD19 expression in a relapsed CD19− sample LK194 (**a**) compared to a typical ALL diagnostic sample (**b**). (**c-e**) CD19 expression in spleen samples from mice transplanted with LK194, harvested 10 weeks post transplant. The green line shows cells labelled with B-cell surface markers CD10, CD34, CD58, human-specific CD45 and mouse-specific CD45. This acts as a control for the pink histogram, which shows cells labelled with these same cell surface markers but with the addition of CD19. Only human cells are shown. See also Supplementary Figures 5 and 7. **(f**, **g)** CD19 expression in spleens of mice transplanted with samples from Mouse 2 (**f**) or Mouse 3 (**g**). See also Supplementary Figure 8 (**h**). Gel electrophoresis image of PCR products to detect the expression of CD19 exons 1–3, using cDNA from LK194 mouse samples. Arrows show expression of CD19 transcript containing exons 1–3 (403 bp) and absence of the variant that skips exon 2 (expected size 137 bp). SEM cells were used as a CD19^+^ control. NTC, non-template control.
