# Supplementary material for: BCP-ALL blasts are not dependent on CD19 expression for leukaemic maintenance
Source: Leukemia. 2016 Apr 8;30(9):1920–3. doi: 10.1038/leu.2016.64 (PMC4950966; doi:10.1038/leu.2016.64)
Supplement: Supplementary Information [file leu201664x1.docx]

**Supplementary materials and methods**

***Cell culture***

The 293T lentiviral producer cell line was cultured in DMEM (HEPES modification), supplemented with 10% FBS (GIBCO, Life Technologies, Paisley, UK), 4 mM L-glutamine and 1 mM sodium pyruvate. The BCP-ALL cell lines SEM, REH, 697 and the feeder cell line M2-10B4 were grown in RPMI 1640 medium containing 10% FBS. Primografts were maintained in StemSpan SFEM (StemCell Technologies, Manchester, UK), supplemented with 20% FBS, 1% Penicillin/Streptomycin, 20 ng/ml recombinant IL-3 (R & D Systems, Abingdon, UK), 10 ng/ml recombinant IL-7 (R & D Systems). Human MSC were obtained from bone marrow of a total hip replacement sample using Ficoll density gradient centrifugation and grown in low glucose DMEM supplemented with 20% FBS, 2 nM L-glutamine, 1% Pen/Strep and 8 ng/ml FGF-basic Recombinant Human (GIBCO). Cell proliferation was assessed by counting using the Trypan Blue exclusion assay. Doubling times (Td) were calculated using the following formula: Td(days) = ln(2)/x, where x is the slope of a plot of ln(cell number) against time(d), as in Figure 1a. SEM, REH, 697 and 293T cell lines were acquired from DSMZ (Braunschweig, Germany). All cell culture media and supplements were acquired from Sigma Aldrich (Poole, Dorset, UK), unless otherwise specified.

***Patient samples:***

The patient derived material was collected as part of the initial diagnostic investigation of patients. It was collected, stored and used with written informed consent according to approvals given by the local institutional review boards and the Declaration of Helsinki. Samples were retrieved from Newcastle Haematological BioBank under the generic BioBank approval given by the Newcastle & North Tyneside Ethics Committee (REC reference number: 07/H0906/109). Human MSC samples were collected following appropriate consent and according to approval given by the Newcastle and North Tyneside 1 Research Ethics Committee (REC Reference Number: 09/H0906/72). ALL samples were transplanted into NOD/LtSz-scid IL-2Rγ null (NSG) mice by intrafemoral injection. Engraftment was monitored by collecting blood samples from the tail vein. All work was conducted in accordance with the Home Office Project Licence PPL60/4552.

***Lentiviral vectors and lentivirus production***

The lentiviral transduction of BCP-ALL cell lines was performed using the Tet-On® pTRIPZ inducible lentiviral system allowing induced expression of shRNAmir in the presence of doxycycline. BCP-ALL primografts were transduced with the pGIPZ lentiviral construct, modified to replace the CMV promoter with a spleen focus forming virus (SFFV) promoter. The BCP-ALL cell lines were transduced with two different lentiviral constructs, each targeting a specific region of the open reading frame of the *CD19* gene (CD19 I: Clone ID V2THS_150334; CD19 II: Clone ID V3THS_399245). As a control vector we used an shRNA targeting the fusion gene *RUNX1/ETO*, which is not found in any of the BCP-ALL cell lines or BCP-ALL primografts used. All pTRIPZ and pGIPZ constructs were purchased from GE Life Sciences (Little Chalfont, UK).

Lentivirus was produced based on a standard protocol from D.Trono’s Laboratory of virology and genetics (EPFL, Lausanne, Switzerland). 293T cells were plated in 100 mm culture dishes to achieve 30-40% confluence on the day of transfection. Calcium phosphate precipitation was used to transfect the cells with pMD2.G envelope plasmid, pCMVΔR8.91 packaging plasmid and pTRIPZ/pGIPZ transfer plasmid. After a medium change on the following day, lentivirus was collected 72-96 hours after initial transduction by ultracentrifugation. BCP-ALL cell lines and primografts were transduced as described previously ([Bomken*, et al* 2013](#_ENREF_1)). BCP-ALL cell lines were assessed for successful transduction with pTRIPZ constructs after 4 days of doxycycline induction (1µg/ml) by detecting tRFP using flow cytometry. Transduction of primografts with pGIPZ constructs was assessed by detecting tGFP expression. Successfully transduced BCP-ALL cell lines were selected and maintained in medium containing puromycin (1 µg/ml) to achieve at least 80% tRFP positive cells. BCP-ALL primografts were selected in puromycin (0.5-0.75 µg/ml) and purified using Lymphoprep^TM^ (Axis Shield, Dundee, UK).

***Flow cytometry***

The knockdown of CD19 was determined by staining with CD19-APC (SJ25C1) antibody (BD Biosciences, Oxford, UK). Up to 1 x10^6^ stained cells were washed twice in PBS (GIBCO) containing 0.2% BSA (Roche, West Sussex, UK) (PBSA) and analysed using a FACSCalibur flow cytometer (BD Biosciences). The immunophenotype of the CD19^-^ relapse BCP-ALL patient sample (LK194) was determined by staining with CD10-PECy7, CD34-PerCP, CD19-APCH7, CD38-FITC, CD45-BD Horizon 500C, CD20-PE, CD22-APC and CD123-BV 421 (all BD Biosciences) and analysed using a FACSCanto II flow cytometer (BD Biosciences). Mouse tail bleed samples were labelled as whole blood with CD10-PE, CD34-PerCP, CD19-APC, CD58-FITC, hCD45-APC H7 and mCD45-PeCy7 (all BD Biosciences). Following ammonium chloride red cell lysis, cells were washed twice in PBSA and analysed using a FACSCanto II flow cytometer.

***Competitive assays on co-culture systems***

Following 7 days of doxycycline induction, equal mixtures of untransduced and transduced BCP-ALL cells were seeded at low cell numbers (10^4^ cells/ml in 20ml) in 2% FBS containing medium on M2-10B4 feeder cells. After a medium change every 3 to 4 days cells were harvested by trypsinisation after 7 days, replated under the previously described conditions on fresh feeder cells and finally collected on day 13. At every seeding step the maintenance of tRFP expression was examined using flow cytometry to determine the ratio of wild-type and transduced cells.

Transduced BCP-ALL primografts were cultured on human mesenchymal stem cells (hMSC) in a 24- or 48- well plate, harvested by trypsinisation when confluent and passaged on fresh hMSC. In the competitive assay, the ratio of untransduced and transduced primografts was assessed by detection of tGFP expression using flow cytometry.

***Polymerase chain reaction (PCR)***

DNA and RNA were extracted using DNeasy Blood & Tissue kit and RNeasy Mini Kit respectively (both Qiagen). cDNA was produced from RNA using RevertAid H Minus First Strand cDNA Synthesis Kit (Thermo Fisher Scientific). PCR was carried out using Phusion High-Fidelity PCR Master Mix with HF Buffer (New England Biolabs) as per the manufacturer’s instructions. The following primers were used: CD19 Exon 1 F: GAGAGTCTGACCACCATGCC; CD19 Exon 3 R: CCTAGGTCCGAAACATTCCAC. Products were run on a 2% agarose gel. Products were extracted from the gel using QIAquick Gel Extraction Kit (Qiagen) and sent for Sanger sequencing (Source Bioscience, Nottingham, UK).

**References:**

Bomken, S., Buechler, L., Rehe, K., Ponthan, F., Elder, A., Blair, H., Bacon, C.M., Vormoor, J. & Heidenreich, O. (2013) Lentiviral marking of patient-derived acute lymphoblastic leukaemic cells allows in vivo tracking of disease progression. *Leukemia,* 27, 718-721.

**Supplementary figures:**


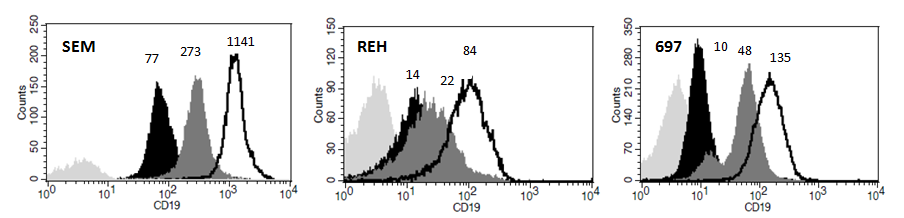


**RUNX1/ETO + dox**

**CD19 I + dox**

**CD19 II + dox**

**Unstained**

**Supplementary Figure 1. CD19 knockdown in BCP-ALL cell lines**

BCP-ALL cell lines were assessed for CD19 knockdown after 5 days of doxycycline (dox) induction by flow cytometry. Graphs show knockdown achieved with two shRNA constructs (CD19 I and CD19 II) in comparison to unstained cells and cells transduced with a control shRNA plasmid (RUNX1/ETO). Numbers represent the CD19-APC geometric mean of fluorescence.

**
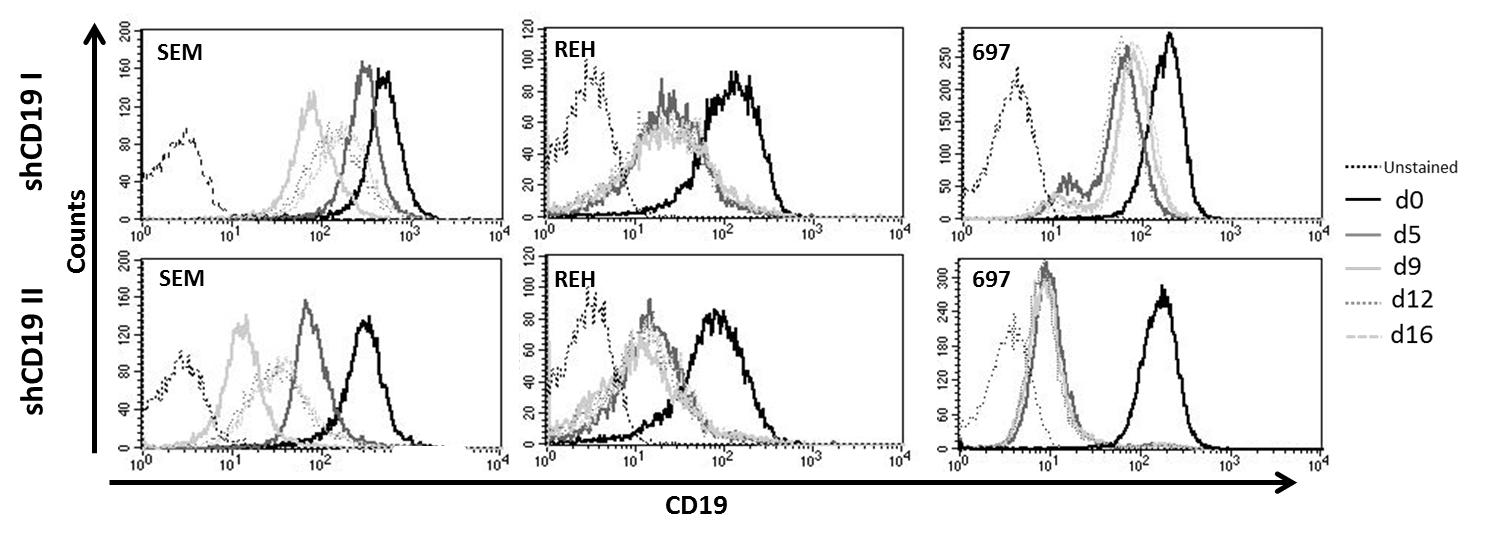
**

**Supplementary Figure 2. Time course of CD19 knockdown in BCP-ALL cell lines**

Histograms show time course of CD19 knockdown with shCD19 I/ II constructs over 16 days in BCP-ALL cell lines. The knockdown of CD19 was monitored at several timepoints (day 0, 5, 9, 12, 16) and was constantly present at the same level in REH and 697 cell line. Transduced SEM cells with both shCD19 I/II constructs showed their lowest knockdown on day 5, which was maximally increased on day 9 and finally slightly stabilized on day 12 and 16. Throughout all timepoints the best knockdown of CD19 surface expression was achieved with the shCD19 II construct.


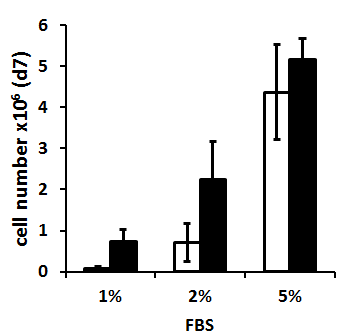

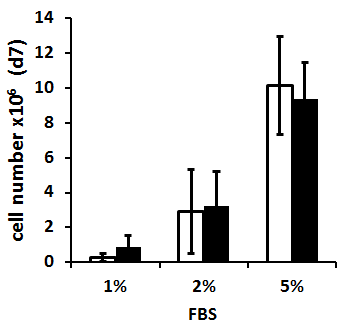

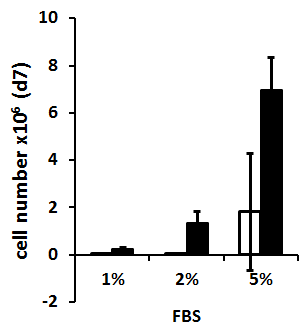


**697**

**REH**

**SEM**

**without feeders with feeders**

**Supplementary Figure 3. Proliferation of wild-type BCP-ALL cell lines at low seeding density with decreasing concentrations of FBS in the absence and presence of feeder cells**

SEM and REH cells proliferated normally in 5% FBS, whereas 697 cells were slightly feeder dependent. SEM and 697 cells failed to proliferate in 1% or 2% FBS, but were able to grow on feeder cells. REH cells did not develop feeder dependence to the same extent and showed similar proliferation in the absence and presence of feeder cells. Graph shows mean of three independent experiments with bars indicating standard error of the mean.

**
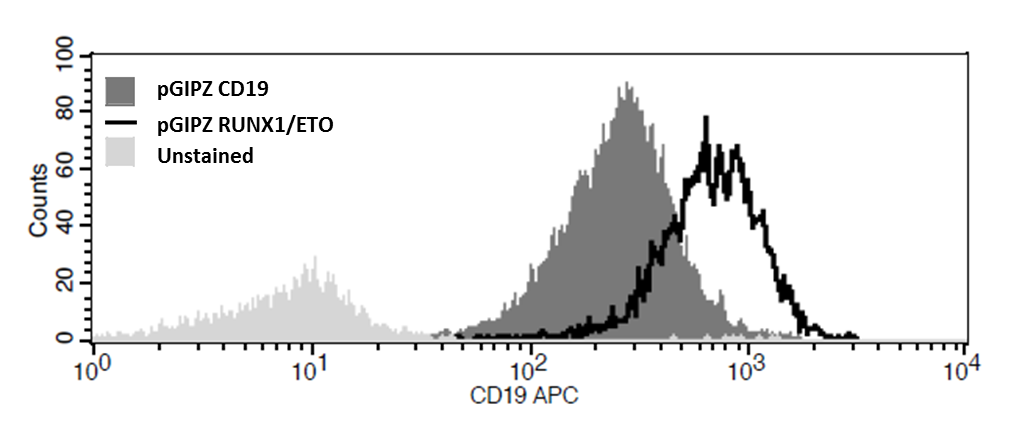
**

**Supplementary Figure 4. CD19 knockdown in CD19 positive high risk BCP-ALL primograft; L707**

An approximately 3 fold reduction in CD19 surface expression was achieved, based on comparing the geometric mean of CD19-APC signal of the control vector pTRIPZ-RUNX1/ETO (658.7) to the pTRIPZ-CD19 (248.1) on day 24.

**
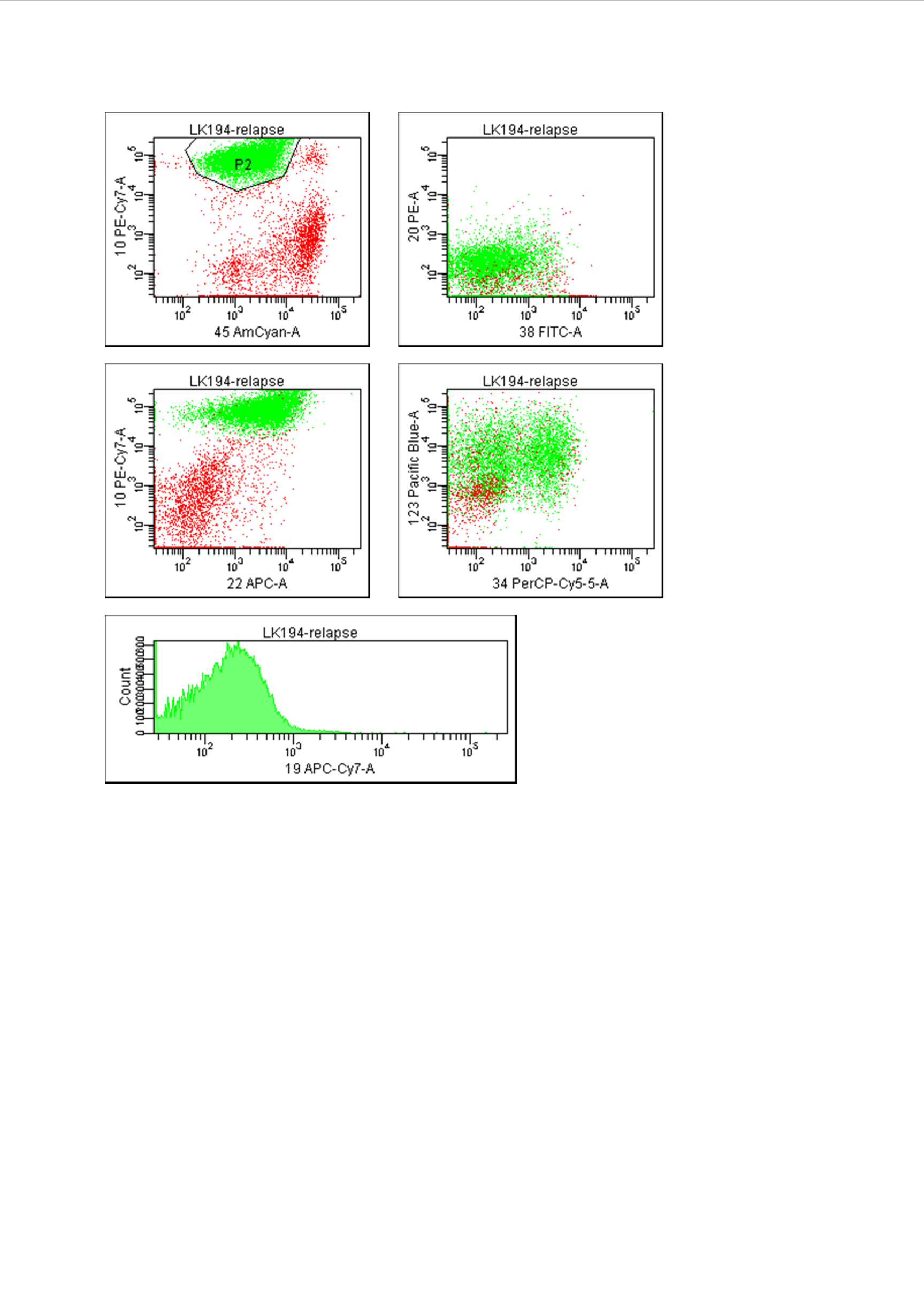
**

**Supplementary Figure 5.**

Immunophenotype of CD19 negative relapsed ALL sample LK194, staining for CD10, CD45, CD22, CD20, CD38, CD123, CD34 and CD19. P2 represents the leukaemic relapse blasts.


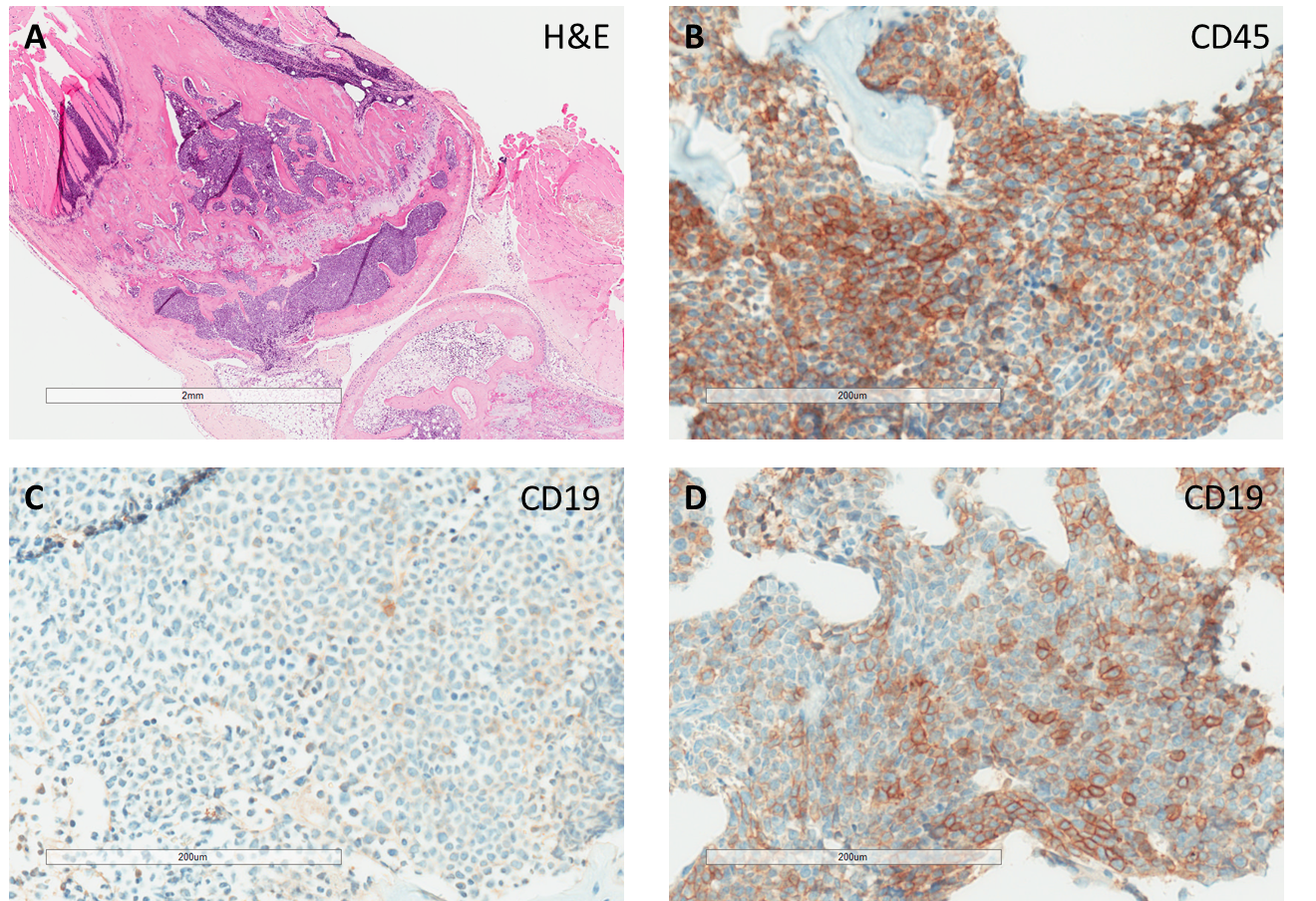


**Supplementary Figure 6.**

Images of paraffin sections from femurs of mice transplanted with sample LK194. A: Haematoxylin & Eosin stained section from Mouse 2 (see Figure 2), 2x magnification. B: CD45 stained section from Mouse 2 (20x magnification). C,D: CD19 stained sections from Mouse 1 (C) and Mouse 2 (D) (20x magnification).


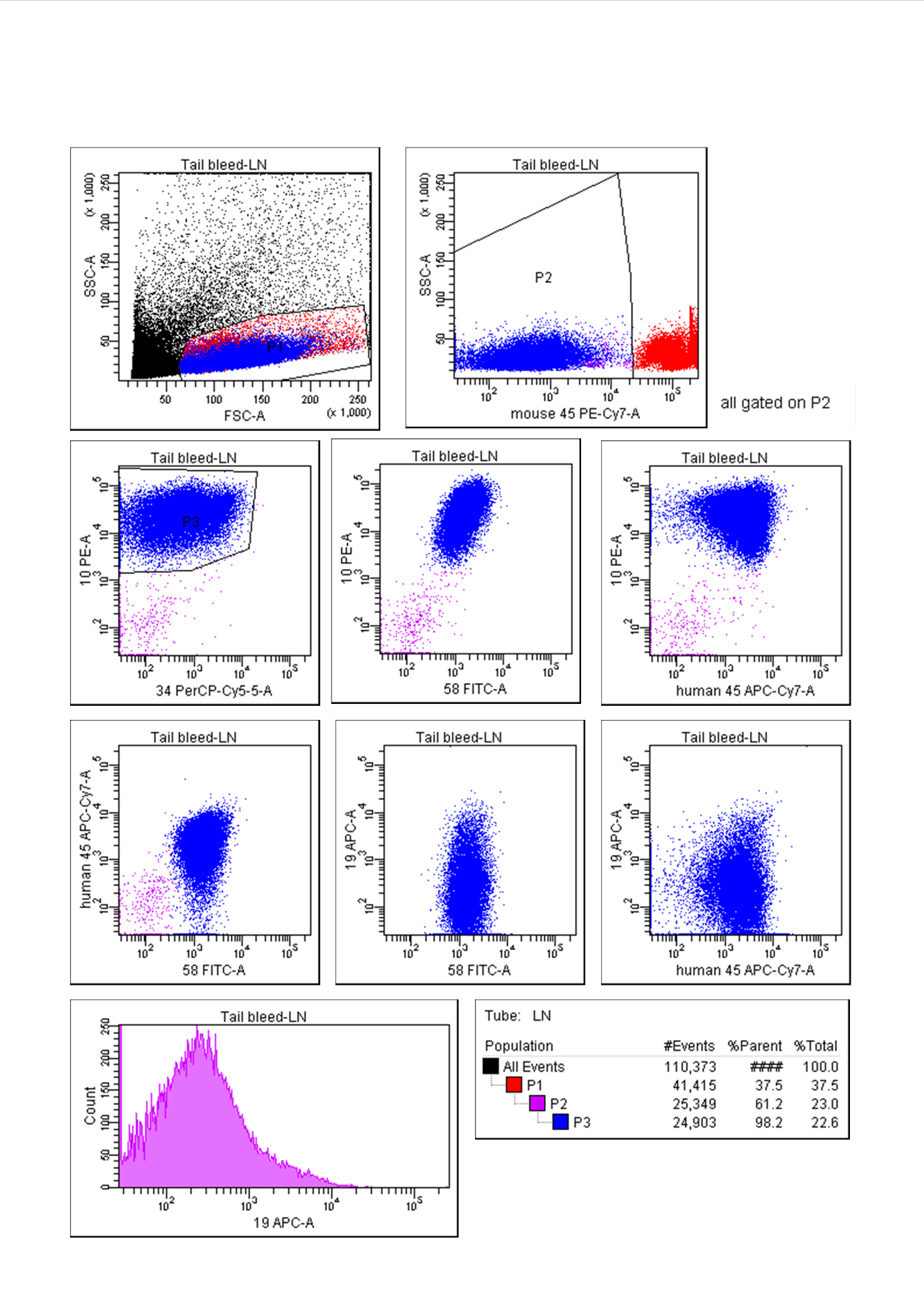


**Supplementary Figure 7.**

FACS analysis of tail vein blood from mouse transplanted with CD19 negative relapsed ALL sample LK194. Area P2 represents human cells with area P3 (blue coloured) representing leukaemic blasts. The histogram of CD19 expression shows all cells in area P2


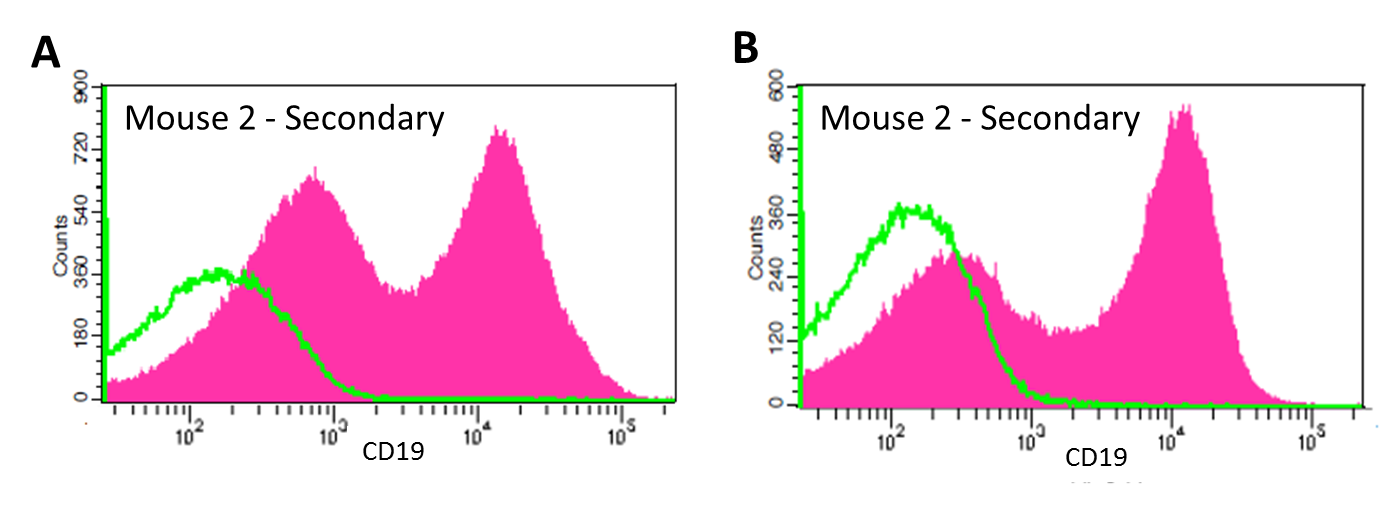


**Supplementary Figure 8.**

CD19 expression in spleens of additional mice transplanted with samples from Mouse 2 (see Figure 2).


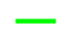
 cells labelled with surface markers CD10, CD34, CD58, human specific CD45 and mouse specific CD45. Only human cells shown.


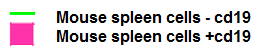
 cells labelled with surface markers CD19, CD10, CD34, CD58, human specific CD45 and mouse specific CD45. Only human cells shown.

CD19_LK194_LN AGGCCCCTGCCTGCCCCAGCATCCCCTGCGCGAAGCTGGGTGCCCCGGAGAGTCTGACCA

CD19_REF AGGCCCCTGCCTGCCCCAGCATCCCCTGCGCGAAGCTGGGTGCCCCGGAGAGTCTGACCA

CD19_SEM AGGCCCCTGCCTGCCCCAGCATCCCCTGCGCGAAGCTGGGTGCCCCGGAGAGTCTGACCA

************************************************************

CD19_LK194_LN CCATGCCACCTCCTCGCCTCCTCTTCTTCCTCCTCTTCCTCACCCCCATGGAAGTCAGGC

CD19_REF CCATGCCACCTCCTCGCCTCCTCTTCTTCCTCCTCTTCCTCACCCCCATGGAAGTCAGGC

CD19_SEM CCATGCCACCTCCTCGCCTCCTCTTCTTCCTCCTCTTCCTCACCCCCATGGAAGTCAGGC

************************************************************

CD19_LK194_LN CCGAGGAACCTCTAGTGGTGAAGGTGGAAGAGGGAGATAACGCTGTGCTGCAGTGCCTCA

CD19_REF CCGAGGAACCTCTAGTGGTGAAGGTGGAAGAGGGAGATAACGCTGTGCTGCAGTGCCTCA

CD19_SEM CCGAGGAACCTCTAGTGGTGAAGGTGGAAGAGGGAGATAACGCTGTGCTGCAGTGCCTCA

************************************************************

CD19_LK194_LN AGGGGACCTCAGATGGCCCCACTCAGCAGCTGACCTGGTCTCGGGAGTCCCCGCTTAAAC

CD19_REF AGGGGACCTCAGATGGCCCCACTCAGCAGCTGACCTGGTCTCGGGAGTCCCCGCTTAAAC

CD19_SEM AGGGGACCTCAGATGGCCCCACTCAGCAGCTGACCTGGTCTCGGGAGTCCCCGCTTAAAC

************************************************************

CD19_LK194_LN CCTTCTTAAAACTCAGCCTGGGGCTGCCAGGCCTGGGAATCCACATGAGGCCCCTGGCCA

CD19_REF CCTTCTTAAAACTCAGCCTGGGGCTGCCAGGCCTGGGAATCCACATGAGGCCCCTGGCCA

CD19_SEM CCTTCTTAAAACTCAGCCTGGGGCTGCCAGGCCTGGGAATCCACATGAGGCCCCTGGCCA

************************************************************

CD19_LK194_LN TCTGGCTTTTCATCTTCAACGTCTCTCAACAGATGGGGGGCTTCTACCTGTGCCAGCCGG

CD19_REF TCTGGCTTTTCATCTTCAACGTCTCTCAACAGATGGGGGGCTTCTACCTGTGCCAGCCGG

CD19_SEM TCTGGCTTTTCATCTTCAACGTCTCTCAACAGATGGGGGGCTTCTACCTGTGCCAGCCGG

************************************************************

CD19_LK194_LN GGCCCCCCTCTGAGAAGGCCTGGCAGCCTGGCTGGACAGTCAATGTGGAGGGCAGCGGGG

CD19_REF GGCCCCCCTCTGAGAAGGCCTGGCAGCCTGGCTGGACAGTCAATGTGGAGGGCAGCGGGG

CD19_SEM GGCCCCCCTCTGAGAAGGCCTGGCAGCCTGGCTGGACAGTCAATGTGGAGGGCAGCGGGG

************************************************************

CD19_LK194_LN AGCTGTTCCGGTGGAATGTTTCGGACCTAGGTGGCCTGGGCTGTGGCCTGAAGAACAGGT

CD19_REF AGCTGTTCCGGTGGAATGTTTCGGACCTAGGTGGCCTGGGCTGTGGCCTGAAGAACAGGT

CD19_SEM AGCTGTTCCGGTGGAATGTTTCGGACCTAGGTGGCCTGGGCTGTGGCCTGAAGAACAGGT

************************************************************

CD19_LK194_LN CCTCAGAGGGCCCCAGCTCCCCTTCCGGGAAGCTCATGAGCCCCAAGCTGTATGTGTGGG

CD19_REF CCTCAGAGGGCCCCAGCTCCCCTTCCGGGAAGCTCATGAGCCCCAAGCTGTATGTGTGGG

CD19_SEM CCTCAGAGGGCCCCAGCTCCCCTTCCGGGAAGCTCATGAGCCCCAAGCTGTATGTGTGGG

************************************************************

CD19_LK194_LN CCAAAGACCGCCCTGAGATCTGGGAGGGAGAGCCTCCGTGTGTCCCACCGAGGGACAGCC

CD19_REF CCAAAGACCGCCCTGAGATCTGGGAGGGAGAGCCTCCGTGTCTCCCACCGAGGGACAGCC

CD19_SEM CCAAAGACCGCCCTGAGATCTGGGAGGGAGAGCCTCCGTGTGTCCCACCGAGGGACAGCC

***************************************** ******************

CD19_LK194_LN TGAACCAGAGCCTCAGCCAGGACCTCACCATGGCCCCTGGCTCCACACTCTGGCTGTCCT

CD19_REF TGAACCAGAGCCTCAGCCAGGACCTCACCATGGCCCCTGGCTCCACACTCTGGCTGTCCT

CD19_SEM TGAACCAGAGCCTCAGCCAGGACCTCACCATGGCCCCTGGCTCCACACTCTGGCTGTCCT

************************************************************

CD19_LK194_LN GTGGGGTACCCCCTGACTCTGTGTCCAGGGGCCCCCTCTCCTGGACCCATGTGCACCCCA

CD19_REF GTGGGGTACCCCCTGACTCTGTGTCCAGGGGCCCCCTCTCCTGGACCCATGTGCACCCCA

CD19_SEM GTGGGGTACCCCCTGACTCTGTGTCCAGGGGCCCCCTCTCCTGGACCCATGTGCACCCCA

************************************************************

CD19_LK194_LN AGGGGCCTAAGTCATTGCTGAGCCTAGAGCTGAAGGACGATCGCCCGGCCAGAGATATGT

CD19_REF AGGGGCCTAAGTCATTGCTGAGCCTAGAGCTGAAGGACGATCGCCCGGCCAGAGATATGT

CD19_SEM AGGGGCCTAAGTCATTGCTGAGCCTAGAGCTGAAGGACGATCGCCCTGCCAGAGATATGT

********************************************** *************

CD19_LK194_LN GGGTAATGGAGACGGGTCTGTTGTTGCCCCGGGCCACAGCTCAAGACGCTGGAAAGTATT

CD19_REF GGGTAATGGAGACGGGTCTGTTGTTGCCCCGGGCCACAGCTCAAGACGCTGGAAAGTATT

CD19_SEM GGGTAATGGAGACGGGTCTGTTGTTGCCCCGGGCCACAGCTCAAGACGCTGGAAAGTATT

************************************************************

CD19_LK194_LN ATTGTCACCGTGGCAACCTGACCATGTCATTCCACCTGGAGATCACTGCTCGGCCAGTAC

CD19_REF ATTGTCACCGTGGCAACCTGACCATGTCATTCCACCTGGAGATCACTGCTCGGCCAGTAC

CD19_SEM ATTGTCACCGTGGCAACCTGACCATGTCATTCCACCTGGAGATCACTGCTCGGCCAGTAC

************************************************************

CD19_LK194_LN TATGGCACTGGCTGCTGAGGACTGGTGGCTGGAAGGTCTCAGCTGTGACTTTGGCTTATC

CD19_REF TATGGCACTGGCTGCTGAGGACTGGTGGCTGGAAGGTCTCAGCTGTGACTTTGGCTTATC

CD19_SEM TATGGCACTGGCTGCTGAGGACTGGTGGCTGGAAGGTCTCAGCTGTGACTTTGGCTTATC

************************************************************

CD19_LK194_LN TGATCTTCTGCCTGTGTTCCCTTGTGGGCATTCTTCATCTTCAAAGAGCCCTGGTCCTGA

CD19_REF TGATCTTCTGCCTGTGTTCCCTTGTGGGCATTCTTCATCTTCAAAGAGCCCTGGTCCTGA

CD19_SEM TGATCTTCTGCCTGTGTTCCCTTGTGGGCATTCTTCATCTTCAAAGAGCCCTGGTCCTGA

************************************************************

CD19_LK194_LN GGAGGAAAAGAAAGCGAATGACTGACCCCACCAGGAGATTCTTCAAAGTGACGCCTCCCC

CD19_REF GGAGGAAAAGAAAGCGAATGACTGACCCCACCAGGAGATTCTTCAAAGTGACGCCTCCCC

CD19_SEM GGAGGAAAAGAAAGCGAATGACTGACCCCACCAGGAGATTCTTCAAAGTGACGCCTCCCC

************************************************************

CD19_LK194_LN CAGGAAGCGGGCCCCAGAACCAGTACGGGAACGTGCTGTCTCTCCCCACACCCACCTCAG

CD19_REF CAGGAAGCGGGCCCCAGAACCAGTACGGGAACGTGCTGTCTCTCCCCACACCCACCTCAG

CD19_SEM CAGGAAGCGGGCCCCAGAACCAGTACGGGAACGTGCTGTCTCTCCCCACACCCACCTCAG

************************************************************

CD19_LK194_LN GCCTCGGACGCGCCCAGCGTTGGGCCGCAGGCCTGGGGGGCACTGCCCCGTCTTATGGAA

CD19_REF GCCTCGGACGCGCCCAGCGTTGGGCCGCAGGCCTGGGGGGCACTGCCCCGTCTTATGGAA

CD19_SEM GCCTCGGACGCGCCCAGCGTTGGGCCGCAGGCCTGGGGGGCACTGCCCCGTCTTATGGAA

************************************************************

CD19_LK194_LN ACCCGAGCAGCGACGTCCAGGCGGATGGAGCCTTGGGGTCCCGGAGCCCGCCGGGAGTGG

CD19_REF ACCCGAGCAGCGACGTCCAGGCGGATGGAGCCTTGGGGTCCCGGAGCCCGCCGGGAGTGG

CD19_SEM ACCCGAGCAGCGACGTCCAGGCGGATGGAGCCTTGGGGTCCCGGAGCCCGCCGGGAGTGG

************************************************************

CD19_LK194_LN GCCCAGAAGAAGAGGAAGGGGAGGGCTATGAGGAACCTGACAGTGAGGAGGACTCCGAGT

CD19_REF GCCCAGAAGAAGAGGAAGGGGAGGGCTATGAGGAACCTGACAGTGAGGAGGACTCCGAGT

CD19_SEM GCCCAGAAGAAGAGGAAGGGGAGGGCTATGAGGAACCTGACAGTGAGGAGGACTCCGAGT

************************************************************

CD19_LK194_LN TCTATGAGAACGACTCCAACCTTGGGCAGGACCAGCTCTCCCAGGATGGCAGCGGCTACG

CD19_REF TCTATGAGAACGACTCCAACCTTGGGCAGGACCAGCTCTCCCAGGATGGCAGCGGCTACG

CD19_SEM TCTATGAGAACGACTCCAACCTTGGGCAGGACCAGCTCTCCCAGGATGGCAGCGGCTACG

************************************************************

CD19_LK194_LN AGAACCCTGAGGATGAGCCCCTGGGTCCTGAGGATGAAGACTCCTTCTCCAACGCTGAGT

CD19_REF AGAACCCTGAGGATGAGCCCCTGGGTCCTGAGGATGAAGACTCCTTCTCCAACGCTGAGT

CD19_SEM AGAACCCTGAGGATGAGCCCCTGGGTCCTGAGGATGAAGACTCCTTCTCCAACGCTGAGT

************************************************************

CD19_LK194_LN CTTATGAGAACGAGGATGAAGAGCTGACCCAGCCGGTCGCCAGGACAATGGACTTCCTGA

CD19_REF CTTATGAGAACGAGGATGAAGAGCTGACCCAGCCGGTCGCCAGGACAATGGACTTCCTGA

CD19_SEM CTTATGAGAACGAGGATGAAGAGCTGACCCAGCCGGTCGCCAGGACAATGGACTTCCTGA

************************************************************

CD19_LK194_LN GCCCTCATGGGTCAGCCTGGGACCCCAGCCGGGAAGCAACCTCCCTG---GGGTCCCAGT

CD19_REF GCCCTCATGGGTCAGCCTGGGACCCCAGCCGGGAAGCAACCTCCCTGGCAGGGTCCCAGT

CD19_SEM GCCCTCATGGGTCAGCCTGGGACCCCAGCCGGGAAGCAACCTCCCTG---GGGTCCCAGT

*********************************************** **********

CD19_LK194_LN CCTATGAGGATATGAGAGGAATCCTGTATGCAGCCCCCCAGCTCCGCTCCATTCGGGGCC

CD19_REF CCTATGAGGATATGAGAGGAATCCTGTATGCAGCCCCCCAGCTCCGCTCCATTCGGGGCC

CD19_SEM CCTATGAGGATATGAGAGGAATCCTGTATGCAGCCCCCCAGCTCCGCTCCATTCGGGGCC

************************************************************

CD19_LK194_LN AGCCTGGACCCAATCATGAGGAAGATGCAGACTCTTATGAGAACATGGATAATCCCGATG

CD19_REF AGCCTGGACCCAATCATGAGGAAGATGCAGACTCTTATGAGAACATGGATAATCCCGATG

CD19_SEM AGCCTGGACCCAATCATGAGGAAGATGCAGACTCTTATGAGAACATGGATAATCCCGATG

************************************************************

CD19_LK194_LN GGCCAGACCCAGCCTGGGGAGGAGGGGGCCGCATGGGCACCTGGAGCACCAGGTGATCCT

CD19_REF GGCCAGACCCAGCCTGGGGAGGAGGGGGCCGCATGGGCACCTGGAGCACCAGGTGATCCT

CD19_SEM GGCCAGACCCAGCCTGGGGAGGAGGGGGCCGCATGGGCACCTGGAGCACCAGGTGATCCT

************************************************************

CD19_LK194_LN CAGGTGGCCAGCCTGGATCTCCTCAAGTCCCCAAGATTCACACCTGACTCTGAAATCTGA

CD19_REF CAGGTGGCCAGCCTGGATCTCCTCAAGTCCCCAAGATTCACACCTGACTCTGAAATCTGA

CD19_SEM CAGGTGGCCAGCCTGGATCTCCTCAAGTCCCCAAGATTCACACCTGACTCTGAAATCTGA

************************************************************

CD19_LK194_LN AGACCTCGAGCAGATGATGCCAACCTCTGGAGCAATGTTGCTTAGGATGTGTGCATGTGT

CD19_REF AGACCTCGAGCAGATGATGCCAACCTCTGGAGCAATGTTGCTTAGGATGTGTGCATGTGT

CD19_SEM AGACCTCGAGCAGATGATGCCAACCTCTGGAGCAATGTTGCTTAGGATGTGTGCATGTGT

************************************************************

CD19_LK194_LN GTAAGTGTGTGTGTGTGTGTGTGTGTGTATACATGCCAGTGACACTTCCAGTCCCCTTTG

CD19_REF GTAAGTGTGTGTGTGTGTGTGTGTGTGTATACATGCCAGTGACACTTCCAGTCCCCTTTG

CD19_SEM GTAAGTGTGTGTGTGTGTGTGTGTGTG---------------------------------

***************************

CD19_LK194_LN TATTCCTTAAATAAACTCAATGAGCTCTTCCAATCC------------

CD19_REF TATTCCTTAAATAAACTCAATGAGCTCTTCCAATCCTAAAAAAAAAAA

CD19_SEM ------------------------------------------------

**Supplementary Figure 9:**

Comparison of cDNA sequence from CD19- primograft LK194 (Mouse 1) and SEM cell line with a reference (REF) (NCBI NM_001178098.1).
